# The Role of National Specialist Societies in Influencing Transformational Change in Low-Middle Income Countries – Reflections on the Model of Implementation for a National Endoscopy Training Programme in Bangladesh

**DOI:** 10.2147/CEG.S297667

**Published:** 2021-03-22

**Authors:** Neil Hawkes, Umakant Dave, Mesbah Rahman, Dafydd Richards, Mahmud Hasan, A H M Rowshon, Faruque Ahmed, M Masudur Rahman, M G Kibria, Phedra Dodds, Bethan Hawkes, Stuart Goddard, Imdadur Rahman, Peter Neville, Mark Feeney, Gareth Jenkins, Keith Lloyd, Krish Ragunath, Cathryn Edwards, Simon D Taylor-Robinson

**Affiliations:** 1 Department of Gastroenterology, Cwm Taf Morgannwg University Health Board, Llantrisant, South Wales, UK; 2 British Society of Gastroenterology Central Office, London, UK; 3 Department of Gastroenterology, Swansea Bay University Health Board, Swansea, UK; 4 Office of the Central Secretariat, Bangladesh Gastroenterology Society, Dhaka, Bangladesh; 5 Office of Central Secretariat, Gastroliver Foundation, Dhaka, Bangladesh; 6 Department of Gastroenterology, Sheikh Russel National Gastroliver Institute and Hospital, Dhaka, Bangladesh; 7 Department of Endoscopy Nursing, Office of the JAG GRS Team, Powys Health Board, Brecon, UK; 8 Office of the Wales Cancer Network Pathway, Welsh Cancer Network, Cardiff, UK; 9 Welsh Institute of Minimal Access Therapy, Cardiff MediCentre, Welsh Institute for Minimal Access Therapy, Cardiff University, Cardiff, UK; 10 Department of Gastroenterology, Southampton NHS Trust, Southampton, UK; 11 Department of Gastroenterology and Liver Medicine, Torbay and South Devon NHS Foundation Trust, Devon, UK; 12 Faculty of Health and Life Sciences, Medicine, Swansea University, Swansea, UK; 13 Office of the Provost, Faculty of Health Sciences, Bentley Campus, Curtin Medical School, Curtin University, Perth, Western Australia, Australia; 14 Department of Surgery and Cancer, Imperial College London, London, UK

**Keywords:** endoscopy training, Bangladesh, training programmes, gastroenterology training

## Abstract

The British Society of Gastroenterology (BSG) and the Bangladesh Gastroenterology Society (BGS) have collaborated on an endoscopy training programme, which has grown up over the past decade from a small scheme borne out of the ideas of consultant gastroenterologists in Swansea, South Wales (United Kingdom) to improve gastroenterology services in Bangladesh to become a formalised training programme with broad reach. In this article, we document the socioeconomic and historical problems that beset Bangladesh, the current training needs of doctors and how the BSG-BGS collaboration has made inroads into changing outcomes both for gastroenterologists in Bangladesh, but also for the populations they serve.

## Introduction

Bangladesh is the seventh most populous country on earth with a total area of 144,000 km^2^ (land area 133,000 km^2^) and population density of 1064 persons per km^2^ (compared to 48 for the world, 124 for Asia and 345 for India).^1^,^2^ Bangladesh governmental policy to introduce universal healthcare coverage is unlikely to be achieved by 2030 without increases in public funding for health care, introduction of a national health insurance system and expansion of community-based clinics in rural areas.^3^,^4^

### Health Needs in Bangladesh

**Figure 1:**
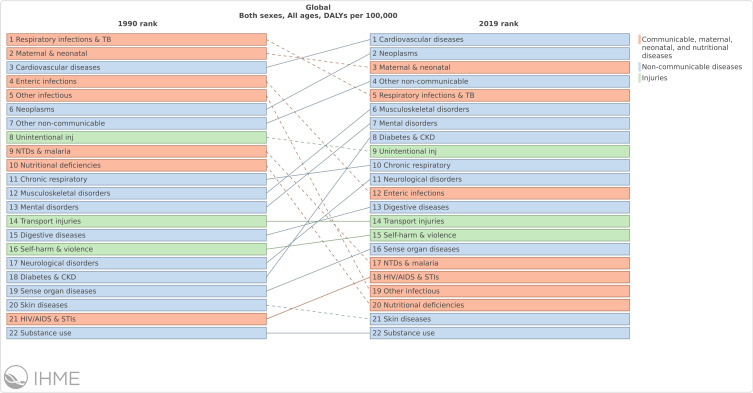
Changing pattern of causes of mortality for the population of Bangladesh (all ages, both sexes) between 1990 and 2017.

Changing health trends in Bangladesh have mirrored those reported globally from LMICs in the past three decades, with an observed fall in deaths due to communicable diseases, but a rise in non-communicable disease.^5^ This has prompted implementation of global policy change to address burdens on healthcare systems and disease prevention strategies.^6^ These changes are illustrated in Figure 1.

Chronic diseases such as ischaemic heart disease, stroke, diabetes, chronic obstructive lung disease and lung cancer are major causes of mortality in a modernising Bangladesh society. Gastrointestinal (GI) diseases, however, continue to be a common cause of morbidity and mortality. Acute diarrhoeal illnesses due to enteric infections remain one of the top ten causes of death in rural communities, but with increased Westernization and migration into urban centres, rising death rates from GI cancers (oesophageal, gastric, colorectal, pancreatic and liver) and cirrhosis-related mortality are being seen. All-cause mortality from cancer in Bangladesh is predicted to increase and environmental issues, such as arsenic poisoning in the water supply, contributing to increase population risk, need to be addressed.^7^,^8^

Systematic reviews of strategies to reduce GI-related cancer support vaccination against hepatitis B to reduce hepatocellular cancer, eradication of *Helicobacter pylori* to reduce gastric cancer and endoscopy for the early detection of colorectal cancer.^9^ Endoscopy is also important in treating patients with gastrointestinal bleeding and in the management of pancreaticobiliary pathology.

### Gastroenterology Training

Gastroenterology is a relatively new speciality in Bangladesh, first described in 1979.^10^ Endoscopy services are mainly provided by gastroenterologists or hepatologists, with a smaller number of surgeons performing endoscopy. A postgraduate degree in Gastroenterology is offered by the Bangladesh College of Physicians and Surgeons (BCPS) as a Fellowship in Gastroenterology, and by Bangabandhu Sheikh Mujib Medical University (BSMMU) as a MD (Doctor of Medicine) in Gastroenterology. Both the Fellowship and the MD degrees are of 5 years duration, following a 1-year internship. Doctors have historically learnt endoscopic skills “on the job”, completing an apprenticeship model of training, aiming to perform “sufficient” procedures, but without any specific certification standards or quality metrics being applied to this process.

This contrasts with the competency-based endoscopy certification process in the UK, overseen by the Joint Advisory Group in GI Endoscopy (JAG).^11^ Trainees in the UK must complete hands-on training courses, have their performance reviewed using Direct Observation of Procedural Skills (DOPS) forms, enter evidence of procedures performed on to an electronic portfolio system (JAG endoscopy training system [JETS e-portfolio]) and complete a final summary assessment by at least two consultant trainers who have not participated in their training. The tools used in the assessment process have been independently validated to provide a robust measure of trainee performance,^12^,^13^ and allow trainees completing training pathways to meet national performance standards for endoscopic practice.^14–16^

### The UK-Bangladesh Link

In 2013, supported by a small International Grant from the British Society of Gastroenterology (BSG), a visiting team of clinicians from Swansea visited Dhaka and began to provide training courses, based on the JAG model, to gastroenterologists in Bangladesh. In this paper, we describe the stages of project evolution leading to a specific memorandum of understanding (MOU) between two national specialist societies and two Universities. We detail the sustainable programme developments arising from this model of intervention, review the ethical dimensions of the partnership and provide reflections on how the intervention model has supported implementation of an e-portfolio and assessment tools developed in the West for adaptation into an LMIC setting, in this case to implement a National Endoscopy Training Programme.

## Methods

### Needs Assessment Phase

The BSG has supported international projects since 2006 when its international grant programme was launched. Since 2018, a more rigorous process of project planning, objective setting and evaluation has been demanded of applicants, with alignment to BSG International strategic goals.^17^ Endoscopy-based projects have received the majority of BSG International grant funding, as endoscopy service development is a common goal worldwide and UK teams have established a track record of providing support with training and service improvement.^18–20^

The initial grant application to visit Dhaka arose from a personal connection between the project lead and doctors working at Shaheed Suhrawardy Medical College Hospital. The Bangladesh Gastroenterology Society (BGS) was actively involved and supported this initial training program which was held at Dhaka Medical College and Hospital. The initial communicated training need was for training in endoscopic retrograde cholangiopancreatography (ERCP). Needs assessment is a critical phase of effective educational interventions and ensures that learning objectives and training methodologies are appropriate.^21^ However, an accurate assessment can be difficult to achieve when planning is being done at a distance. While the team managed to deliver live case demonstration and teaching, the teaching environment was at times far from ideal and some of the visiting faculty found this stressful and difficult.

Ahmed and colleagues have highlighted the health workforce crisis in Bangladesh reporting approximately five physicians and two nurses per 10,000, with a ratio of nurse to physician being only 0.4, leading to inappropriate skill-mix and inequitable distribution between urban and rural regions.^22^ There have been wider calls for policy makers to create an enabling environment for the female workforce, working in a traditionally patriarchal society, to serve the needs and priorities of the health system.^23^ Recognising this training need, the visiting team provided endoscopy nurse training, addressing not only technical skills development, but also nurse empowerment and team-working principles, while respecting local culture.

A more detailed needs-assessment could only be formulated after the initial visit had been fully evaluated (Table 1).

**Table 1:** Identified Positive Aspects and Learning Gaps Following the Index Project Visit

Positive Aspects of Index Visit	Identified Learning Gaps
Strong motivation to improve endoscopy services	Attempting to perform skills beyond competency
Upper GI diagnostic endoscopy widely performed	Training needed for lower GI and sub-specialties
Access to endoscopic equipment reasonable	Mapping of endoscopy services needed
BGS national society keen to support improvement	Lack of structured multi-disciplinary training system

In response to the identified learning gaps, the BSG project team set out the next steps to lay the foundations for further development:

1. A commitment to return visits to Bangladesh to support the aspiration to improve endoscopy service development.
2. Working with the BGS to perform a survey of endoscopy units providing services across Bangladesh.
3. Developing multi-disciplinary training that would support the development of both endoscopist and endoscopy nurse skills.
4. Setting up of a charity (Gastroenterology Training Academy, UK registered charity no. 1,150,282) to support fund-raising activities that would allow future teams of clinical and academic staff to visit Bangladesh to support the aims of the project.

## Results

### Subsequent Collaboration

Over the next 3 years, supported by charitable donation, visiting teams provided further skills-based training, using ex-vivo simulation training models, in upper gastrointestinal (GI) endoscopy, therapeutic endoscopy (GI haemostasis techniques) and ERCP.

In 2016 a nationwide survey was sent to all 197 endoscopy centres in Bangladesh (with responses from 195 [99%]);^24^ 12% were public endoscopy services, with 88% privately run endoscopy centres; 52% were run as diagnostic centres or clinics, with 48% run in a hospital setting.

Gastroscopy was performed in all centres responding. Lower GI endoscopy was less commonly performed. Sixty-five per cent of centres performed colonoscopy – in most centres this is performed with an endoscopy assistant inserting the scope shaft – while 35% performed flexible sigmoidoscopy. Sub-specialty endoscopy is less common – ERCP was performed in 13% of centres, endoscopic ultrasound (EUS) in 2% of centres. The overall numbers of endoscopies reported in the survey in 2016 were: gastroscopy 356,251; colonoscopy 51,252; flexible sigmoidoscopy 10,824; ERCP 4123 and EUS 291. Most procedures were performed in a single procedure room, without sedation and without dedicated recovery areas, 38% of centres were run by a single endoscopist. Ninety-seven per cent of centres reported having the facility to make a computerised report with photos and 92% of centres kept records. Only 18% of centres carried out any audit of clinical practice. Trainees were only present in 5% of the endoscopy centres – these tended to be the larger government-funded, hospital-based centres.^24^

This initial stage of the project was hugely important in cementing trust and mutual respect between the host and visiting teams, through repeated visits, contributions of visiting faculty to the annual scientific meeting of the BGS, and endoscopy training courses. This relationship provided the context for a repeated needs analysis, performed in conjunction with the BGS Executive, which also reflecting on service development needs for Bangladesh. This concluded that to achieve substantive, larger-scale change, a more sustainable training model on a national scale, supported by government policy would be needed.

### Strategic Planning Phase and the Involvement of the British Society of Gastroenterology

The BGS identified key strategic goals to facilitate developing their national endoscopic service:

1. Renewed calls to the Bangladesh Government via the Health Ministry and Prime Minister’s office for investment into a Gastroenterology and Hepatology Specialist Institute that could act as a central training hub for endoscopy.
2. Involvement of the BSG via their international committee.
3. Implementation in Bangladesh of methodologies developed in the UK to support training and service improvement developments in endoscopy.
4. A widening of the project goals to include the development of hepatology services, and increased collaboration between academic and research institutions.

This shift in project focus from training a few individuals from a few local hospitals, to a strategy of national standard setting and inclusion of robust training methodology into a national programme, constituted a major cultural shift within the GI community in Bangladesh and therefore set significant challenges.

More formal engagement from the BSG International Committee resulted from visits of key committee members as part of the Dhaka project teams in 2017 and 2018. BSG international strategy emphasises the need for development of sustainable projects aligned with the vision of providing the best care and achieve the best outcomes for patients with gastrointestinal and liver diseases in settings across the world,^17^ and is aligned to global strategies of other UK-based medical colleges to foster collaboration.^25^,^26^ Strategic agreement was agreed between the two specialist societies – BGS and BSG – to collaborate on service improvement for endoscopy using the Global Rating Scale, a self-reporting tool introduced in the UK to assess specified areas of endoscopy service delivery;^27^ and implementation of a National Endoscopy Training Programme – based on the UK JAG training system.

This was described in a specific memorandum of understanding (MOU) between the BGS and BSG specialist societies, signed by the respective Presidents of the Societies in February 2020 (Table 2). The MOU set out timetabled project development goals, provided access to wider expertise and a mechanism to work with the International Committee of the JAG to introduce an electronic e-portfolio system. The aim is to deliver a competency-based certification process for trainee endoscopists in Bangladesh, which can be bench-marked against international performance standards.

**Table 2:** Key Roles and Elements with the Memorandum of Understanding Between the Two National Specialist Societies (BGS and BSG)

BGS Commitments	BSG Commitments
Identify a site and faculty base capable of acting as a national training centre and ensure adequate resources to support this including technical support for simulation	Provide advice and guidance on the requirements for endoscopy training centres, including the set-up of ex-vivo simulation models
Agree local certification criteria and system to monitor and document the certification process	Provide models for certification processes and advise where local modifications may be appropriate
Agree and implement measurement of key performance indicators for all endoscopists identified as potential trainers	Provide models for measurement of performance standards and performance management frameworks
To agree and implement a version of the GRS tool in all endoscopy units hosting trainee, with a commitment to improve units and support a national endoscopy training network	Provide modifications to the GRS tool and advice on means of improving endoscopy services, with assistance in the ongoing monitoring of units playing an active part in the training network
To provide ongoing training data submissions (based on training data inputs to an electronic portfolio in pilot centres)	BSG and JAG teams to provide training on the implementation of an e-portfolio system to support endoscopy training and supervise data returns
To publish a National Register of Endoscopy Trainees and Trainers	To oversee criteria for programme entry and advice on requirements for trainers
To publish a programme of National Endoscopy Training Courses to support the development of endoscopy trainees on the Training Register (with planned courses matched to demand)	To advise on course content and work with the Bangladesh faculty to standardise and quality assure programmed courses
To publish a programme for the development of endoscopy assistants matched to an endoscopy assistant competency framework	To develop an endoscopy nurse assistant competency framework and monitor the documentation of competency development
To identify Endoscopy Programme Clinical and Administrative Leads and project management team	To arrange monthly project management meetings, monitor progress against project goals, provide support and advice, and review training related data.
To communicate the aims and objective of the National Endoscopy Training Programme project to all stakeholders in Bangladesh	To agree a programme of ongoing visits and contribution to the Annual Conference of the Bangladesh Gastroenterology Society

### Implementation and Monitoring Phase

The current project phase supports the programmed implementation of the MOU. A significant boost has come from government investment to build the Sheikh Russel Gastroliver Institute and Hospital in Dhaka. This comprises a government-owned, specialist hospital to investigate and treat gastroenterological and hepatological conditions and to provide a national hub for endoscopy training. An inaugural training course was conducted here in February 2019 and the hospital is now open for patient care. The BGS leadership team continue to have the support of the Health Ministry of the Bangladesh Government in delivering a National Endoscopy Training Programme. A MOU project management team has access to virtual tools such as Zoom™ and Microsoft Teams™ to maintain contact, share documents and provide training on the use of the e-portfolio and educational training tools. The first 9 months of the project implementation phase comprises a 3-month training period on use and data entry into the e-portfolio, followed by a 6-month period of data entry focused at three sites across Bangladesh. This will build towards wider data entry from sites across Bangladesh and will represent the first documented implementation of a competency-based national endoscopy training programme imported from a Western country into an LMIC environment.

**Figure 2:**
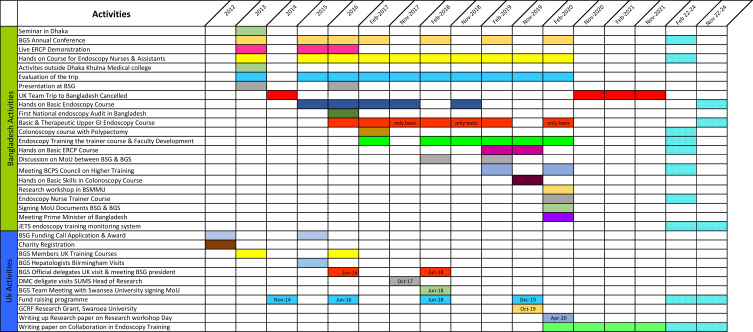
Gantt chart showing the timelines of the collaboration between the United Kingdom and Bangladesh parties to improved endoscopy training.

**Abbreviations:** BGS, Bangladesh Gastroenterology Society; BSG, British Society of Gastroenterology; ERCP, endoscopic retrograde cholangiopancreaticography; MoU, memorandum of understanding; BCPS, Bangladesh College of Physicians & Surgeons; BSMMU, Bangabandhu Sheikh Mujib Medical University; JETS, JAG endoscopy training system; DMC, Dhaka Medical College; SUMS, Swansea University Medical School; GCRF, Global Challenge Research Fund.

Figure 2 collates together all the timelines in the various phases of the project into an easy-to-follow Gantt chart.

## Discussion

### Wider Benefits of Collaboration and Partnership

While the MOU relates to the specific aim of implementing a National Endoscopy Training Programme, parallel strategic objectives and workforce developments are being supported by the foundation of collaborative working between the wider sections of the specialist societies. Swansea University has become a major academic partner, having contributed visiting faculty, and has signed a separate MOU with Dhaka Medical College, to develop and advance medical training, provide opportunities for international student exchange and to increase research collaborations. Faculty members from leading hepatology units in the UK have provided training on core hepatology and contributed to training in research methods, producing a summary of the Proceedings of a GI Research Workshop for publication.^28^

It is acknowledged that more work needs to be done in advancing recognition for the specialised role of the endoscopy nurse in Bangladesh, and in the International Year of the Nurse, the leadership team of the BGS are seeking to work with the Ministry of Health and Chief Nursing Office to review the status and training pathways for nurses working in endoscopy. Core skills development and training the nurse trainer courses have been delivered, but the wider challenges of nurse recruitment in Bangladesh need to be addressed in addition to increasing opportunities for higher training and contributions to leadership and service improvement from the nursing workforce.

### Planned Exit Phase

The basis of the agreement of the MOU between two National Specialist Societies provides a natural mechanism for transfer of responsibility from visiting to home faculty. The pilot implementation phase will test the robustness of training infrastructure and examine the completeness of data entry that is required to support effective training and an evidence base on which to base certification. A 3-year period (with an additional 2-year “buffer period”) to implement learning from the Pilot Phase and inform the roll-out to additional centres is envisaged. This is important to ensure standardised implementation of training methods in every centre where training is taking place; that demand for training courses is met; to quality assure the training provide by local faculty and their own endoscopic performance standards; and ensure that wider environmental standards of Units and team training is in place. There will also be a graduated and planned handover to ensure sound administration and quality assuring the National Endoscopy Training Programme, supported by the UK JAG Administrative Office. Basing implementation on proven, evidence-based tools and standards provides a means for benchmarking outcomes against standards and metrics used in the UK. The ongoing data entry into an electronic database can be monitored at distance to track trainer and trainee participation and provide a robust evidence base to assist the local team in the ongoing task of quality assurance. All costs of ongoing remote support have been built into the programme.

The long-term aim of the programme, via the implementation of a competency-based endoscopy training programme, is to increase the quality of endoscopy in Bangladesh. The introduction of structured JAG training programmes in the UK has been credited as being an important part in improved patient-related outcomes and effectiveness of endoscopy reported in National Audits.^29^ Ensuring effective monitoring of the practice of independent endoscopists in Bangladesh will continue to be a challenge, especially given the more fragmented nature of Health Services in Bangladesh, compared to the relatively standardised National Health Service in the UK.

The World Endoscopy Organisation is providing additional support and assistance to the BGS. Through their centre of excellence programme, there are further incentives for the training centre in Dhaka to achieve further recognition if they can meet all the aims and objectives of the MOU between the two national societies.

The principles of partnership outlined by THET^30^ have been used to test all aspects of the ongoing partnership and to avoid the dangers of voyeuristic “healthcare tourism”. The development of the MOU has underscored the roles and value of each partner organisation. Despite this, reflections from individual project members have confirmed themes identified by Hunt and colleagues,^31^ such as tensions in respect to local values and imposition of western methodologies; local barriers to the provision of adequate care; differing understandings of health, illness and local healthcare systems; a culture of deference; and differences in cultural understanding between local and visiting teams, all of which have been recognisable at various stages. Individual members of the team, especially those visiting Bangladesh for the first time, may perceive these differences most acutely. Project leads, and organisations, need to be proactive in mitigating moral distress.^32^

Bangladesh will be one of the nations most affected if global warming targets of limiting global heating to less than 1·5°C above pre-industrial temperatures, defined in the Paris Agreement, are not met. Sustainable solutions and evidence-based strategies to support “green endoscopy”,^33^ need to be shared and developed in LMIC settings. Visiting teams increasingly need to consider the overall effect of their visit on the environment. One practical contribution to this has been the collection and donation of “out of date” endoscopy accessories and decommissioned endoscopy processor equipment, that can still be safely used, being “recycled” and transported to Bangladesh for clinical use. Over the eight visits the team has made, a considerable amount of equipment has been donated to support patient care.

Finally, the importance of “well-being” is being increasingly recognised and legislated for in Western Societies.^34^ The visiting team has provided teaching on “well-being” and “burn-out” for health-care professionals working within the endoscopy service, and evidence-based reviews of mindfulness techniques as a strategy to support staff and patients. A baseline survey of burnout in gastroenterologists (trainee and senior staff) is being set-up in Bangladesh in collaboration with the visiting team.

## Conclusion

The project partnership between two national specialist societies outlined in this paper provides a concrete example of how specialist medical societies can work together to implement effective change at a national level, building on relationships of trust, built by initial project teams working at local level with support from low-level grant funds. In this project, repeated visits helped to refine a detailed needs assessment and a shared strategic vision of how these needs might be most effectively met. The insights and awareness within the BGS team of the political and infrastructural barriers to changing the culture of endoscopy training and negotiation of a strategic approach likely to achieve change and project delivery within an LMIC context has been invaluable. This project delivers a technological solution to enhance training, but inherent risks and barriers are acknowledged. Literature reviews point to only a minority of projects attempting to use technology employing thorough evaluation. Technology has been successful in LMIC settings in helping to track patients; improve communication and flow of information; register samples and patients; monitor patients or provide reminders of appointments; assist with research; and reduce laboratory or medication-related errors.^35^ Other large-scale trials of the use of technology in Bangladesh, such as mobile phone-based technology to improve health care are being conducted.^36^

Ultimately, healthcare interventions, including those involving international visiting teams, must demonstrate value – for our project, this will be demonstrable improvement in the standard of local endoscopy and through transferable skills gained, benefits to patients from access to better quality endoscopy. While local clinicians, familiar with operating in resource-limited environments, often have an intrinsic sense of value-based healthcare delivery,^37^ they lack the necessary tools to measure outcomes. The adoption of evidence-based training methodologies and tools, adapted for use in LMIC environments and allied to data collection in e-portfolios, provides a path to more robust analysis of outcomes. Specialist societies with expertise in applying these methodologies can provide meaningful help in these areas.

The ability to present a clear vision for delivery and international expertise to support implementation has attracted governmental backing and investment to create an environment for change. The detail of the MOU between the two National Societies has provided both the means to effectively manage this change, with agreed specific project goals and means of project evaluation, and a mechanism for a sustainable hand-over, once the project infrastructure has been effectively tested and the training data inputs confirm self-sustainability. We believe that this is a model which might find wider application, where evidence-based tools that promote high-quality education and service improvement can be transferred effectively into LMIC settings. The exploration of the phases of project evolution and comments on the ethics of international partnership provide a reminder of the timescale needed to plan and conduct sustainable project work and of the continued responsibility of project teams to assess the wider ethical and value-based aspects of project implementation.
